# Exploring the relationship between pain and self-harm thoughts and behaviours in young people using network analysis

**DOI:** 10.1017/S0033291721000295

**Published:** 2022-11

**Authors:** Verena Hinze, Tamsin Ford, Robin Evans, Bergljot Gjelsvik, Catherine Crane

**Affiliations:** 1 Department of Psychiatry, University of Oxford, Oxford, UK; 2 Department of Psychiatry, University of Cambridge, Cambridge, UK; 3 Department of Statistics, University of Oxford, Oxford, UK; 4 Department of Psychology, University of Oslo, Oslo, Norway

**Keywords:** Adolescence, children, network analysis, pain, self-harm thoughts and behaviours, young people

## Abstract

**Background:**

Self-harm thoughts and behaviours (SHTBs) are a serious public health concern in young people. Emerging research suggests that pain may be an important correlate of SHTBs in young people. However, it remains unclear whether this association is driven by the shared association with other correlates of SHTBs. This study used network analysis to delineate the relationship between SHTBs, pain and other correlates of SHTBs in a population-based sample of young people.

**Methods:**

We performed secondary analyses, using data from 7977 young people aged 5–16 years who participated in the British Child and Adolescent Mental Health Survey in 2004. We used χ^2^ tests and network analysis to examine the complex interplay between SHTBs, pain and other correlates of SHTBs, including psychiatric disorders, childhood trauma, stressful life events, parental distress, family dysfunction, peer problems and inhibitory control deficits.

**Results:**

Pain was associated with a doubled risk of SHTBs, and likewise, SHTBs were associated with a doubled risk of pain. Furthermore, network analysis showed that although pain was significantly associated with all measured correlates of SHTBs, except family dysfunction, pain was most strongly associated with SHTBs, after accounting for these measured correlates.

**Conclusions:**

To the best of our knowledge, this is the first study to utilise network analysis to provide novel insights into the complex relationship between SHTBs, pain and other known correlates of SHTBs in young people. Results suggest that pain is an independent correlate of SHTBs. Future research should aim to identify underlying mechanisms.

## Introduction

Suicide is a major health concern in young people (Patton et al., 2009; World Health Organisation [WHO], 2018). However, for each suicide, there are many more young people who think about suicide and self-harm or engage in self-harm (‘suicidality’; Bridge, Goldstein, & Brent, 2006; WHO, 2018). The term ‘self-harm’ concerns a variety of behaviours surrounding intentional self-injury or self-poisoning, irrespective of suicidal intent (Hawton, Saunders, & O'Connor, 2012). Self-harm is an important risk factor for future suicide even several years following the act of self-harm (Hawton et al., 2020). The prevalence of self-harm thoughts and behaviours (SHTBs) substantially increases during early adolescence (Hawton et al., 2012; Morgan et al., 2017; Nock et al., 2013), with ~30% of adolescents, reporting lifetime self-harm thoughts and 10% a history of self-harm behaviours (Evans, Hawton, Rodham, & Deeks, 2005). Given the potentially fatal outcome, it is vital to explore correlates that may be associated with, and might ultimately contribute to, SHTBs in young people to inform prevention strategies.

Recently, considerable progress has been made in understanding suicidal risk (O'Connor & Nock, 2014). Commonly studied correlates of SHTBs and suicide in young people fall within three domains, namely: (i) socio-demographics and educational factors, (ii) stressful life events and family adversity, including parental distress, family dysfunction and peer problems and (iii) personality and mental-health factors, including cognitive deficits (see online Supplementary Table S1; Hawton et al., 2012; Mars et al., 2019).

Empirical research and theoretical models of suicidality in adults suggest pain to be an important correlate of (non-)suicidal SHTBs (Joiner, 2005; Klonsky & May, 2015; O'Connor & Kirtley, 2018; Racine, 2018; Tang & Crane, 2006; Van Orden et al., 2010). Headaches (8–83%) and abdominal pain (4–53%) are the most common pain complaints in young people, followed by musculoskeletal pain (4–40%) and back pain (12–24%), with also a considerable increase in prevalence rates in early adolescence (King et al., 2011; Martin, McGrath, Brown, & Katz, 2007). As the experience of pain comprises both the physical sensations and emotional response to it (Price, 2000; Soltani, Kopala-Sibley, & Noel, 2019), prolonged and severe pain may considerably affect an individual's mood (Noel, Groenewald, Beals-Erickson, Gebert, & Palermo, 2016; Soltani et al., 2019). A recent systematic review provides support for a relationship between pain and (non-)suicidal SHTBs in adolescence (Hinze et al., 2019). However, the mechanisms underlying this observed association are unclear and likely to be complex (see Klonsky & May, 2015; Lewcun et al., 2018). As known correlates of SHTBs (Hawton et al., 2012) greatly overlap with correlates of chronic pain in young people (e.g. psychopathology, stressful life events, family functioning, parental distress and peer relationships; McKillop and Banez, 2016), it is important to understand whether the potential association between pain and SHTBs holds, after accounting for such correlates.

Most previous research has employed regression analyses to demonstrate the independent association between pain and SHTBs, after controlling for demographics and depression (see Fuller-Thomson, Hamelin, & Granger, 2013; Van Tilburg, Spence, Whitehead, Bangdiwala, & Goldston, 2011). Network analysis can extend these traditional methods by offering the means to quantify and visually display the potential relationship between pain and SHTBs, in the context of a range of other variables included in the network (Borsboom & Cramer, 2013; De Beurs, 2017). The online Supplement S1 provides further information on network analysis.

Although there is an increasing application of network analysis in suicide research, most research so far has explored symptom networks (see De Beurs, van Borkulo, & O'Connor, 2017). Alternatively, network analysis can be used to decode the interrelationship between known correlates of SHTBs (‘risk networks’; see De Beurs, 2017). For instance, network analysis has been applied to explore the interplay and unique contribution of risk and resilience factors for suicidal ideation in veterans (Simons et al., 2019). To date, network research has predominantly focussed on adult populations, so little is known about the complex interplay and the unique association between known correlates, including pain, and SHTBs among young people.

The present study utilised network analysis to delineate the relationship between SHTBs, pain and other correlates of SHTBs in a population-based sample of young people aged 5–16 years. Here, the term ‘self-harm thoughts and behaviours’ (SHTBs) will be used to broadly refer to passive or active SHTBs of unknown intent, given the low prevalence rates of SHTBs in the current sample of young people. We hypothesised that reports of pain would be associated with reports of SHTBs, and that this association would remain significant, after accounting for other measured and shared correlates of SHTBs and pain (i.e. psychiatric disorders, childhood trauma, stressful life events, parental distress, family dysfunction, peer problems and inhibitory control deficits) and regularisation for weak associations. Furthermore, we explored how SHTBs and pain may relate to these other measured correlates of SHTBs in young people. To our knowledge, research on the association between pain and SHTBs in young people has solely focussed on the adolescent years. Given the potential developmental differences (Peyre et al., 2017; Sarkar et al., 2010), we performed a series of sensitivity analyses to explore whether the relationship between SHTBs, pain and these other measured correlates of SHTBs may be different between children (5–10 years) and adolescents (10–16 years).

## Methods

### Participants and ethical considerations

We performed secondary data analyses, using the British Child and Adolescent Mental Health Survey (BCAMHS) in 2004. The Medical Research Ethics Committee granted ethical approval for the initial survey. The current analyses required no formal permission other than data access permission. The survey sample and methods have been described in detail elsewhere (see Green, McGinnity, Meltzer, Ford, & Goodman, 2005). The final sample consisted of 7977 children aged 5–16 years. Information, analysed in the current study, was collected predominantly from parents, but some data was obtained from a teacher nominated by the family, and adolescents aged 11 years or older (Green et al., 2005).

### Measures

#### Self-harm thoughts and behaviours

Three standardised questions were used to assess current or past SHTBs (see Green et al., 2005), which were collapsed into one ‘SHTB’ variable. Parents and adolescents, aged 11 years and older (*n* = 4052 of which *n* = 3123 (77%) had available data on SHTBs), were asked whether the child: (a) talked about deliberately harming or hurting himself/herself, over the last 4 weeks, (b) tried to harm or hurt himself/herself over the last 4 weeks and (c) ever tried to harm or hurt himself/herself, over the whole of his/her lifetime. The variable ‘SHTB’ was treated as a dichotomous variable, referring to the presence or absence of self-harm thoughts and/or behaviours; coded as present, if it was reported by either or both informants, and absent if neither endorsed self-harm thoughts and/or behaviour items. The inter-rater agreement between parents and adolescents was ‘fair’ [Cohen's kappa = 0.33; bootstrapped 95% CI (0.25–0.40); sensitivity = 51%; specificity = 95%; McHugh, 2012]. Specifically, the use of adolescent data led to an additional identification of 132 young people with SHTBs, where parents reported SHTBs to be absent (*n* = 3617). Where parental data was missing (*n* = 277), the use of adolescent data led to an identification of 17 young people with and 144 young people without SHTBs, whilst for 116 young people, data on SHTBs was missing for both informants.

#### Pain

Parents were asked to indicate whether their child was experiencing any health problems or conditions, using the question: ‘Here is a list of health problems or conditions which some children or young people may have. Please can you tell me whether NAME CHILD has…’ followed by a list of medical conditions amongst which (a) stomach/digestive problems or abdominal/tummy pain and (b) migraines or severe headaches (Green et al., 2005). These different pain locations were collapsed into one dichotomous ‘pain’ variable, referring to the presence or absence of pain conditions.

#### Other correlates of SHTBs

The present data set (Green et al., 2005) included the following known and shared correlates of SHTBs and pain: psychiatric disorders (combined information from parents, teachers and young people, aged 11 years and older, using the Development and Well-Being Assessment (DAWBA); Goodman, Ford, Richards, Gatward, & Meltzer, 2000), childhood trauma (information from parents, using the ‘post-traumatic stress disorder’ module of the DAWBA; Goodman et al., 2000), stressful life events (information from parents, using a list of ten stressful life events; Goodyer, Wright, & Altham, 1990), parental distress (parental report, using the General Health Questionnaire; (GHQ-12); Goldberg et al., 1997), family dysfunction (information from parents, using the McMaster Family Assessment Device; Miller, Epstein, Bishop, & Keitner, 1985), peer problems and inhibitory control deficits (information from parents using the ‘peer problem’ and ‘hyperactivity/inattention’ subscales of the Strength and Difficulties Questionnaire; Goodman, 1997). For the purpose of the planned analyses, all correlates were dichotomised to reflect the presence or absence of each correlate. The assessment of each correlate is further described in online Supplement S2.

### Statistical analyses

#### Data description

Descriptive statistics were used to explore sample characteristics, and χ^2^ analyses were used to investigate whether pain and SHTBs were significantly associated, using SPSS version 25 (IBM Corporation, 2017). Bootstrapping was used to explore the stability of the parameter estimates, using 1000 bootstrap samples, with a *p* value of 0.05 reflecting statistical significance. All subsequent analyses were performed in the software package R, version 3.6.1 (R Core team, 2019).

#### Network estimation

We computed a series of weighted network models to examine the potential relationships between variables and depict the magnitude of these associations (McNally, 2016). The network estimation was based on the *Ising model* (Ising, 1925; Kindermann & Snell, 1980), using a series of nodewise logistic regression analyses to estimate the network parameters from our binary data (Van Borkulo et al., 2014). We decided to use binary data, focussing on the overall relationship between SHTBs and pain in young people, because of the item-based assessment of SHTBs and pain in the current study, and in the interest of modelling the relationship between pain and SHTBs, whilst accounting for a multitude of other measured correlates of SHTBs, which would lower the statistical power (Epskamp, Borsboom, & Fried, 2018). Consistent with recommended practices (Van Borkulo et al., 2014), we estimated the regularised partial correlation network using the R package *IsingFit* (Van Borkulo, Epskamp, & Robitzsch, 2016) and the method *elasso* (Van Borkulo et al., 2014), which is a regularisation technique that sets small estimates to zero, thereby eliminating spurious edges.

Using the packages *qgraph* (Epskamp, Cramer, Waldorp, Schmittmann, & Borsboom, 2012) and *networktools* (Jones, 2020), we retrieved information on the node's strength and expected influence [one-step (EI1) and two-step (EI2) expected influence; McNally, 2016; Robinaugh, Millner, & McNally, 2016] to explore the importance of each node in the network. Consistent with recommended procedures (Heeren, Jones, & McNally, 2018), we tested for the influence of restricted variability on the node's importance (Terluin, De Boer, & De Vet, 2016) by computing the Pearson correlations between the node's variance and centrality indices. For more details, please see online Supplement S1.

## Results

### Participant characteristics

Table 1 describes the characteristics of the whole sample, and for children and adolescents separately. The sample of 7977 young people had an equal gender distribution (girls: *n* = 3866; 48.5%) and a mean age of 10.54 years, s.d. = 3.4. Most young people were White (*n* = 6920; 86.8%), and in most families, at least one parent was employed (*n* = 6601; 82.8%). Current or past SHTBs were identified in 399 (5%) young people; 64 (16.0%) of whom also reported pain. Of the remaining 7306 (91.6%) young people without SHTBs, who had also available data on pain (*n* = 7230), 584 (8.1%) young people reported pain. Across the total sample, pain was reported for 702 (8.8%) young people. Of those with pain, who also had available data on SHTBs (*n* = 648), 64 (9.9%) young people reported SHTBs. In contrast, in the remaining 7163 young people without pain, and with available data on SHTBs (*n* = 6980), 334 (4.8%) young people reported SHTBs.

**Table 1.** Sample characteristics of the whole study sample in 2004 (*N* = 7977)

Variable	Children (*n* = 3235)	Adolescents (*n* = 4742)	Total (*N* = 7977)
Demographics			
Age, *M* (s.d.)	7.03 (1.43)	12.93 (1.97)	10.54 (3.40)
Girls, *n* (%)	1578 (48.8)	2288 (48.2)	3866 (48.5)
SHTBs			
SHTBs (either one or both), *n* (%)^a^	75 (2.3)	324 (6.8)	399 (5.0)
Self-harm thoughts (no behaviour), *n* (%)^b^	30 (0.9)	51 (1.1)	81 (1.0)
Self-harm behaviour (with thoughts), *n* (%)^a^	6 (0.2)	45 (0.9)	51 (0.6)
Self-harm behaviour (without thoughts), *n* (%)^a^	39 (1.2)	228 (4.8)	267 (3.3)
Correlates of SHTBs			
Psychiatric disorder (DSM-IV), *n* (%)	240 (7.4)	524 (11.1)	764 (9.6)
Childhood trauma (PTSD module), *n* (%)^c^	530 (16.4)	1171 (24.7)	1701 (21.3)
Stressful life events (SLE module), *n* (%)^d^	1662 (51.4)	2908 (61.3)	4570 (57.3)
Parental distress (GHQ-12), *n* (%)^e^	638 (19.7)	1109 (23.4)	1747 (21.9)
Family dysfunction, *n* (%)^f^	511 (15.8)	838 (17.7)	1349 (16.9)
Peer problems, reported by the parent, *n* (%)^g^	321 (9.9)	542 (11.4)	863 (10.8)
Inhibitory control deficits, reported by the parent, *n* (%)^h^	703 (21.7)	815 (17.2)	1518 (19.0)
Physical pain, *n* (%)^i^	212 (6.6)	490 (10.3)	702 (8.8)

^a^Missing data for 272 participants (3.4%), ^b^missing data for 274 participants (3.4%), ^c^missing data for 187 participants (2.3%), ^d^missing data for 203 participants (2.5%), ^e^missing data for 241 participants (3.0%), ^f^missing data for 276 participants (3.5%), ^g^missing data for 42 participants (0.5%), ^h^missing data for 52 participants (0.7%) and ^i^missing data for 112 participants (1.4%).

*Note*. Children = aged 5–9 years; adolescents = aged 10–16 years; self-harm thoughts and behaviours, self-harm thoughts, self-harm behaviour = composite scores, incl. parental report and/or young person report.

### The association between pain and SHTBs

χ^2^ analyses revealed a significant association between pain and SHTBs; pain was associated with a doubled risk of SHTBs, and SHTBs were associated with a doubled risk of pain (OR 2.18, bootstrap 95% CI 1.64–2.83; *φc* = 0.064, bootstrap 95% CI 0.037–0.093).

### Network analyses

**Fig. 1.**
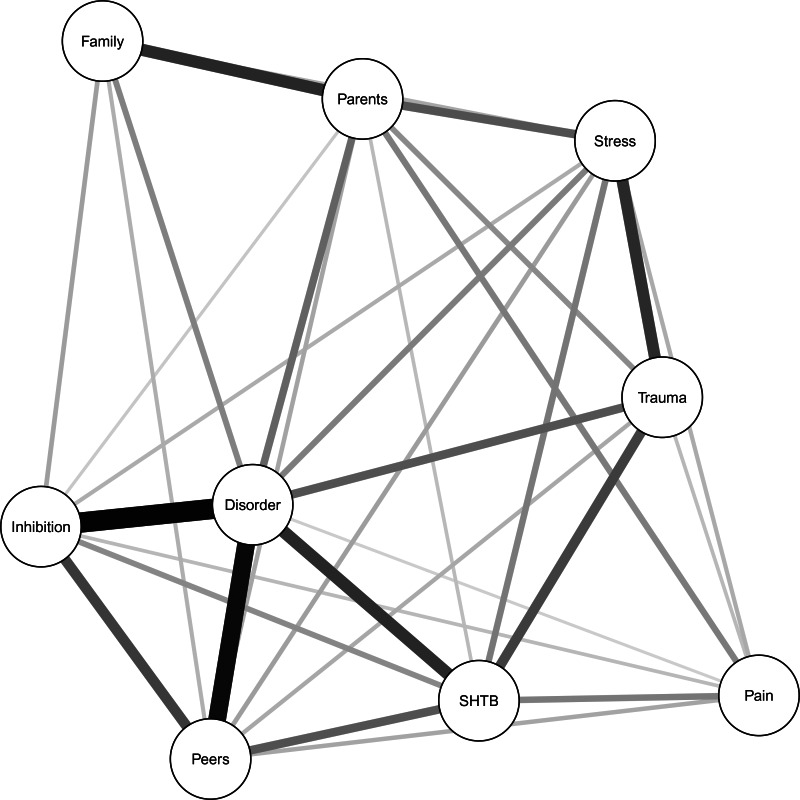
Regularised partial correlation network of the whole sample (*N* = 7513). Each circle (i.e. node) represents a separate variable in the network. The thickness of the connecting line (i.e. edge) between two nodes represents the strengths of their relationship, after accounting for all other nodes included in the network, with thicker/darker edges representing stronger associations (Borsboom & Cramer, 2013). *Legend:* SHTB, self-harm thoughts and behaviours; Disorder, psychiatric disorder(s); Stress, stressful life event(s); Trauma, childhood trauma; Parents, parental distress; Family, family dysfunction; Peers, peer problems; Inhibition, inhibitory control deficits.

The regularised partial correlation network consisted of 9 nodes and 32 non-zero edges (Fig. 1) and showed a significant relationship between pain and SHTBs in the whole study sample, after accounting for all other measured correlates of SHTBs (i.e. psychiatric disorders, childhood trauma, stressful life events, parental distress, family dysfunction, peer problems and inhibitory control deficits) and regularisation for weak associations (Table 2). The wide confidence interval suggests some instability across the 1000 bootstrap estimations, which should, therefore, be interpreted cautiously.

**Table 2.** Weights matrix for the regularised partial correlation network estimation on the whole sample (*N* = 7513)

	SHTB	Disorder	Stress	Trauma	Parents	Family	Peers	Inhibition	Pain
SHTB	–	**0.98**	0.46	**0.77**	0.20	0	**0.64**	0.39	0.46
Disorder		–	0.43	0.64	0.54	0.41	1.47	1.70	0.15
Stress			–	0.94	0.65	0.27	0.30	0.25	0.26
Trauma				–	0.37	0	0.26	0	0.21
Parents					–	0.97	0.28	0.17	0.44
Family						–	0.24	0.30	0
Peers							–	0.81	0.28
Inhibition								–	0.21
Pain									–

*Legend:* SHTB, self-harm thoughts and behaviours; Disorder, psychiatric disorder(s); Stress, stressful life event(s); Trauma, childhood trauma; Parents, parental distress; Family, family dysfunction; Peers, peer problems; Inhibition, inhibitory control deficits.

*Note.* The strongest associations with ‘self-harm thoughts and behaviours’ are highlighted in **bold**, whilst the strongest associations with ‘Pain’ are underlined.

An examination of the edge weights (Table 2) revealed that the variable ‘SHTB’ was significantly associated with all measured correlates of SHTBs, except family dysfunction. The strongest association was revealed with psychiatric disorders, which was supported by the narrow bootstrap confidence interval (online Supplementary Fig. S1), followed by childhood trauma, peer problems, stressful life events and pain. The wider bootstrap confidence intervals suggest the increasing instability of the results (online Supplementary Fig. S1).

Pain was significantly associated with all other measured correlates of SHTBs, except family dysfunction (Table 2). The strongest association for pain was with SHTBs, followed by parental distress. However, the wide bootstrap confidence interval suggests instability in the edge weight estimations (online Supplementary Fig. S1).

**Fig. 2.**
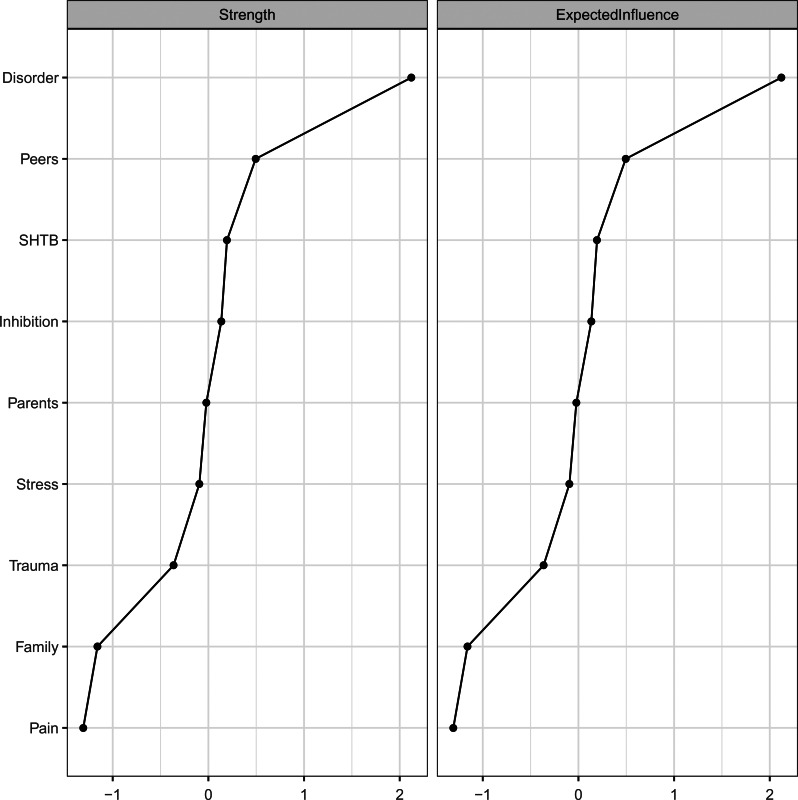
Node-specific centrality indices for the regularised partial correlation network***.*** *Legend:* Disorder, psychiatric disorder(s); Peers, peer problems; SHTB, self-harm thoughts and behaviours; Inhibition, inhibitory control deficits; Parents, parental distress; Stress, stressful life event(s); Trauma, childhood trauma; Family, family dysfunction. Please note, *z* scores rather than raw scores are displayed.

Exploratory analyses highlighted the importance of ‘psychiatric disorders’ as the most influential node in the network, based on all centrality estimates (Fig. 2 and Table 3). Other influential nodes were ‘peer problems’, ‘SHTB’ and ‘inhibitory control deficits’. Noteworthy, ‘pain’ was the least influential node in the network (Fig. 2 and Table 3). The Pearson correlations between node variability and centrality indices were non-significant (strength/EI1: *r* = −0.26, *p* = 0.506; EI2: *r* = −0.30, *p* = 0.438), showing that restricted variability across nodes does not influence conclusions regarding the node's importance (see Heeren et al., 2018; Terluin et al., 2016). Consistent with previous studies (see Heeren et al., 2018), the expected influence indices were highly correlated (*r* = 0.97, *p* < 0.001).

**Table 3.** Centrality analyses for the regularised partial correlation network

	SHTB	Disorder	Stress	Trauma	Parents	Family	Peers	Inhibition	Pain
Strength	3.89	6.33	3.53	3.19	3.62	2.18	4.27	3.82	1.99
Expected influence									
One-step	3.89	**6**.**33**	3.53	3.19	3.62	2.18	4.27	3.82	1.99
Two-step	20.02	**29**.**65**	16.60	16.41	16.12	11.36	23.12	22.10	9.84

*Legend:* SHTB, self-harm thoughts and behaviours; Disorder, psychiatric disorder(s); Stress, stressful life event(s); Trauma, childhood trauma; Parents, parental distress; Family, family dysfunction; Peers, peer problems; Inhibition, inhibitory control deficits.

*Note.* Higher values in **bold** show the most influential nodes.

Finally, we performed a series of exploratory analyses to compare the regularised partial correlation networks between children (aged 5–9 years, *n* = 3079) and adolescents (aged 10–16 years, *n* = 4434; online Supplement S3). These analyses showed that the relationship between pain and ‘SHTB’ was not significant in the networks of both children and adolescents. A statistical comparison of the regularised partial correlation networks of both age groups revealed no significant differences in the overall network structure (children = 14.02 *v.* adolescents = 13.51; *p* = 0.484) and network strength (*p* = 0.852). Significant group differences were revealed for the edges between SHTBs and parental distress (present in children but not in adolescents; *p* = 0.004) and between psychiatric disorders and family dysfunction (present in adolescents but not in children; *p* = 0.018).

## Discussion

We explored the relationship between pain and SHTBs in a population-based sample of young people aged 5–16 years. We found that 5% of young people in this study experienced current or past SHTBs, and 9% of young people reported pain, which is a lower estimate than in similar studies (SHTBs: 10–30%; Evans et al., 2005; pain: 11–38%; King et al., 2011). These findings are likely to relate to the age of our study sample and our assessment of pain and SHTBs, as discussed below. Furthermore, pain was associated with a doubled risk of SHTBs, and similarly, SHTBs were associated with a doubled risk of pain, which is consistent with our first hypothesis and previous research in adolescents (Hinze et al., 2019). Network analysis provided novel insights into the association between pain and SHTBs, by revealing that this commonly observed association (Hinze et al., 2019) holds in young people, after accounting for known correlates of SHTBs, including psychiatric disorders, childhood trauma, stressful life events, parental distress, family dysfunction, peer problems and inhibitory control deficits.

In all networks, the presence of SHTBs was most strongly associated with the presence of psychiatric disorders, which is consistent with previous research, using regression analysis to demonstrate the independent association between pain and SHTBs, after controlling for demographic variables and depression (Fuller-Thomson et al., 2013; Gili et al., 2019; Van Tilburg et al., 2011). Family dysfunction was neither associated with SHTBs nor with pain, which replicates the finding that family dysfunction is only indirectly associated with suicidal thoughts through its effect on psychiatric disorders (Prinstein, Boergers, Spirito, Little, & Grapentine, 2000).

Our exploratory centrality analyses highlighted that ‘psychiatric disorders’ was the most influential node, which may be due to the higher prevalence rates of psychiatric disorders (10%), compared to SHTBs (5%) in our sample, but also by the commonly detected associations between correlates of SHTBs and psychiatric disorders. For instance, childhood trauma and parental distress are correlates of SHTBs, but also of psychiatric disorders (Carr, Martins, Stingel, Lemgruber, & Juruena, 2013; Crawford, Cohen, Midlarsky, & Brook, 2001).

Although pain was found to be the least influential node in the network, it was associated with all measured correlates of SHTBs, except family dysfunction. These correlates of SHTBs, which we found to be associated with pain, greatly overlap with previous research identifying similar correlates of pain in young people (e.g. psychopathology, stressful life events, parental distress and peer relationships; McKillop & Banez, 2016). After these relationships were accounted for in the regularised partial correlation network, we found that pain was most strongly associated with SHTBs. This suggests that any increased risk of SHTBs in young people with pain (see Wang, Juang, Fuh, & Lu, 2007) is not simply the consequence of the shared associations with other correlates. However, the question of direct and indirect influences of pain on SHTBs requires further research.

Furthermore, whilst the current findings suggest that reported pain is associated with an *increased* risk of SHTBs, some individuals who self-harm may report a *decreased* pain perception (i.e. *increased* pain thresholds and tolerance; see Kirtley, O'Carroll, & O'Connor, 2016). One explanation for these inconsistent findings may be the focus on different clinical populations. In young people with chronic pain (i.e. increased pain perception), pain may be associated with SHTBs through feelings of perceived burdensomeness (see Klonsky & May, 2015), given the reliance on support from others to manage the daily life with pain. Other young people may have an innate or acquired, elevated pain tolerance (i.e. decreased pain perception), which, in the context of, for example poor self-regulatory skills, may increase suicidal risk (see Joiner, 2005; Van Orden et al., 2010). Alternatively, there may be a developmental profile to the relationship between pain and SHTBs. Specifically, an increased pain perception may initially enhance the risk of SHTBs, whilst over time self-harm behaviours may lower the perceived pain perception, through processes of habituation (see De Paepe, Williams, & Crombez, 2019), leading to an increased pain tolerance towards certain stimuli. These speculative explanations require further research, with a specific focus on the temporal order and a differentiation between self-harm thoughts and types of self-harm behaviours in young people with significantly increased or decreased pain levels.

### Strengths and limitations

The present study is the first study to investigate the complex association between pain and SHTBs in young people, after accounting for a multitude of empirically supported correlates of SHTBs. Furthermore, the analyses of a large population-based survey, which employed robust methods and validated measures, increases confidence in our findings and their generalisability.

Despite these strengths, our findings should be interpreted in the light of their limitations. In the present study, reports of SHTB in children aged 5–10 years, and all reports of pain, were solely based on parental reports. As parents may be unaware of their child's SHTBs or pain, these reports will reflect the upper end of the severity spectrum. This is consistent with the moderate sensitivity (50.5%) between parental and adolescent reports of SHTBs, as well as the low prevalence rates of both SHTBs and pain compared to previous studies: SHTBs: 5% *v.* 10–30% (Evans et al., 2005); pain: 9% *v.* 11–38% (King et al., 2011). Nevertheless, the assessment of pain included the most common manifestations of pain in young people (King et al., 2011; Martin et al., 2007), and it seems to be frequent and severe pain—which is more likely to be identified via parental reports—that may be most strongly associated with SHTBs in adolescence (Hinze et al., 2019).

In order to address the potentially low sensitivity of identifying SHTBs with parental reports, we have used a combined measure of SHTBs reported by either the parent and/or young people aged 11years and older. This approach revealed a ‘fair’ inter-rater agreement (Cohen's kappa = 0.33; McHugh, 2012). The use of adolescent data led to a further identification of young people for whom SHTBs were not reported by the parents or with missing parental data. This finding is consistent with previous research (Janiri et al., 2020) and highlights possible problems in the disclosure and parental awareness of young peoples' SHTBs, which need to be considered in clinical practice and future research, when assessing SHTBs. Furthermore, as child-level data was only available for young people aged 11 and older, this approach could have led to a greater identification of SHTBs in young people above the age of 11 years, compared to younger children. However, as SHTBs tend to be uncommon in children (Nock et al., 2013) and as we did not find any differences in the relationship between pain and SHTBs between children and adolescents, as well as in the overall network structure and strength, it seems unlikely that the multiple informant approach for adolescents influenced these findings. Of course, we may have lacked the power to detect such effects. The revealed edge differences between children and adolescents should be interpreted with caution.

Furthermore, whilst the assessment of self-harm behaviours referred to the past 4 weeks and the young person's lifetime, the assessment of self-harm thoughts only covered the past 4 weeks, potentially leading to under-identification of young people with self-harm thoughts. This is consistent with the lower prevalence rates of SHTBs in the present study. However, the relatively younger age of our sample compared to previous studies (i.e. age range: 12–20 years: Evans et al., 2005) may also contribute to the differences in prevalence rates, as the prevalence of SHTBs increases from the age of 12 years onwards (Morgan et al., 2017; Nock et al., 2013). Future studies could explore the relationship between pain and SHTBs more thoroughly, by using a comprehensive clinical examination of the presence and absence of SHTBs and pain, with multiple-informant reports of pain and SHTBs and clear timelines to more closely capture all young people, who may experience pain or SHTBs. More reliable identification of young people with pain and SHTBs may lead to greater statistical power to reveal network associations (see Epskamp et al., 2018).

Another limitation refers to the nature of the SHTBs questions. By asking whether the young person has ‘tried to’ self-harm, it is potentially somewhat ambiguous whether the behaviour has actually occurred, as well as the potential intention of the behaviour. Nevertheless, these questions refer to earlier stages of the SHTBs trajectory (e.g. O'Connor & Kirtley, 2018) and may offer some insights as to whether the young person shows early signs of suicidal distress, and hence falls on this trajectory.

Our study used binary variables, which may hide any differences in the network associations that may relate to specific types of SHTBs or pain. For instance, the severity, frequency, chronicity or the impact of pain on the young person's daily life, including physical, affective, cognitive and social functioning may be associated with different manifestations of SHTBs and different levels of medical severity. However, the crude measure of pain in this study did not allow for an exploration of these differential associations. Furthermore, previous work suggests that SHTBs may be associated with shared, as well as distinct risk factors (Klonsky & May, 2014). Despite the theoretical differentiation between SHTBs, we collapsed SHTBs into one variable, as our measures and sample size (and subsequently prevalence rates of SHTBs) were insufficient to support separate analysis and hence the questions whether pain may be differently associated with SHTBs and whether it may increase the transitional probability of moving from self-harm thoughts to self-harm behaviours await further scrutiny.

The current data set included only a subset of empirically supported correlates of SHTBs in young people (Hawton et al., 2012; Mars et al., 2019), which may limit the content validity and impact the network structure (see Hevey, 2018). Other unmeasured correlates may account for the association between pain and SHTBs. For instance, parents may model maladaptive coping responses, which may ultimately influence the development of both pain and SHTBs, if the young person lacks skills to cope effectively in times of adversity (Cousins, Kalapurakkel, Cohen, & Simons, 2015; O'Connor & Kirtley, 2018). Therefore, future studies should explore additional correlates to further elucidate the relationship between pain and SHTBs. In this context, studies may also wish to explore the mechanisms through which pain may be associated with SHTBs in young people (e.g. acquired capability; Joiner, 2005; Van Orden et al., 2010), by computing directed graphs (see Borsboom & Cramer, 2013).

Finally, the cross-sectional study design means that no conclusions can be drawn about the direction of the effects. Future research should explore the complex interactions of pain with known correlates of SHTBs in order to predict SHTBs longitudinally, using time-series network analyses.

### Clinical implications

Our study identified pain as an independent correlate of SHTBs in young people, which is consistent with the previously observed increased vulnerability to (non-)suicidal SHTBs in young people with pain (Dean-Boucher, Robillard, & Turner, 2020; Hinze et al., 2019). Given the doubled prevalence rates of SHTBs in those young people with pain, compared to the overall sample, these findings may be of particular clinical significance. Although we acknowledge that many young people, who report correlates of SHTBs, such as pain, may never develop SHTBs (Franklin et al., 2017), clinical awareness of this increased vulnerability is essential to offer timely help and support (WHO, 2014). Specifically, all paediatric pain clinicians should receive awareness and management training to inform them about the relationship between pain and SHTBs in young people and to help them to identify common precursors of self-harm or suicidal thoughts (e.g. according to leading suicide theories, these precursors involve feelings of hopelessness or being a burden on others; see Klonsky and May, 2015). Young people with chronic pain should be asked directly about SHTBs at clinical appointments (WHO, 2014), particularly given recent work showing that asking about suicide and SHTBs reduced future risk (Blades, Stritzke, Page, & Brown, 2018). Additionally, as young people spend a large proportion of their time in school (Paulus, Ohmann, & Popow, 2016), it would be important to offer similar training to teachers and school staff, who could offer initial support and refer vulnerable pupils to healthcare services, where necessary (WHO, 2014).

## Conclusion

This study is the first to utilise network analysis to provide novel insights into the complex relationship between SHTBs, pain and other correlates of SHTBs in young people (i.e. psychiatric disorders, childhood trauma, stressful life events, parental distress, family dysfunction, peer problems and inhibitory control deficits). Based on the χ^2^ analyses, we found that twice as many young people with pain reported SHTBs and vice versa. Furthermore, network analyses showed that the relationship between pain and SHTBs persists after accounting for other correlates of SHTBs and regularisation for weak associations. As the correlates we controlled for greatly overlap with key correlates of pain in young people, these novel findings suggest that pain may be an independent correlate of SHTBs in young people. Future research should identify mechanisms through which pain may be associated with SHTBs in young people, with the aim to inform the development of effective prevention strategies. Clinically, our findings suggest the need for timely help and support for those young people who experience pain and who may be at risk of developing SHTBs.

## Syntax sharing

The corresponding syntax files will be made available on the Open Science Framework at the time of publication (see https://osf.io/; project name: ‘Network analysis of the association between pain and self-harm thoughts and behaviours in young people’).

## Supplementary material

For supplementary material accompanying this paper visit https://doi.org/10.1017/S0033291721000295. click here to view supplementary material
