# Supplementary material for: Exploring the relationship between pain and self-harm thoughts and behaviours in young people using network analysis
Source: Psychol Med. 2021 Mar 15;52(15):3560–9. doi: 10.1017/S0033291721000295 (PMC9772913; doi:10.1017/S0033291721000295)
Supplement: Supplementary file 1 [file S0033291721000295sup001.pdf]

Supplementary Material for the Manuscript:

Exploring the relationship between pain and self-harm thoughts and behaviours in young people using network analysis

Authors:

Verena Hinze[a], Prof Tamsin Ford[b], Prof Robin Evans[c]

Dr Bergljot Gjelsvik[a,d♦] & Dr Catherine Crane[a♦]

a. Department of Psychiatry, University of Oxford, Oxford, UK
b. Department of Psychiatry, University of Cambridge, Cambridge, UK
c. Department of Statistics, University of Oxford, Oxford, UK
d. Department of Psychology, University of Oslo, Oslo, Norway

♦ shared last authorship

Published in Psychological Medicine (2021)

DOI: https://doi.org/10.1017/S0033291721000295

Corresponding author:

Ms Verena Hinze

University of Oxford, Department of Psychiatry

Warneford Lane, Oxford, OX3 7JX

Email: verena.hinze@psych.ox.ac.uk (alternatively: verena.hinze@web.de)

TableS1. Commonly studied correlates of self-harm thoughts and behaviours and suicide in young people.

**Socio-demographics and educational factors**	**Stressful life events and family adversity**	**Mental-health and personality factors**
Gender (females are at higher risk of suicidal behaviours and males are at higher risk of completed suicides) [a,b]	Adverse childhood experiences/ life events [a,b]	Big-5 personality traits, including lower emotional stability, conscientiousness, and extraversion as well as higher agreeableness [b]
One's sexual orientation [a]	History of physical or sexual abuse [a,b]	Presence of any mental health disorder, particularly depression, anxiety and attention-deficit-hyperactivity disorder [ADHD] [a,b]
Low socioeconomic status [a,b]	Parental separation/ divorce/ death/mental health [a]	Alcohol and drug misuse [a,b]
Restricted educational achievement [a]	Family discord [a]	Body dissatisfaction [b]
Lower IQ [b]	Self-harm in family and friends [a,b] (particularly higher extent of exposure to self-harm) [b]	Low self-esteem [a]
	Peer problems (i.e., bullying and interpersonal difficulties) [a,b]	Impulsivity [a]
		Hopelessness [a,b]
		Perfectionism [a]
		Poor social problem solving [a]

Note. The correlates of self-harm and suicide have been derived from a review exploring correlates of self-harm and death by suicide in adolescents (see Hawton et al., 2012), as well as from the supplementary material of a recent UK-based study (i.e., ALSPAC study) on correlates of (non-)suicidal self-harm thoughts and behaviours in young people (Mars et al., 2019).

a. Risk correlates have been derived from Hawton et al., 2012.
b. Risk correlates have been derived from Mars et al., 2019.

Supplement 1. Extra information on network analyses.

Scientific rationale

Network analysis is a data-driven statistical analysis technique that can estimate and scrutinise complex relationships between different psychological phenomena (e.g., symptoms and correlates of psychopathology; see Borsboom, 2017; Borsboom & Cramer, 2013). In network models, each variable (i.e., commonly symptoms, such as fatigue or low mood in depression) is represented by a node and the connecting edge between two nodes indicates the relationship between two variables, e.g., symptom A (fatigue) and B (low mood; Borsboom & Cramer, 2013). The thickness of the edge between the nodes depends on how strongly they are related, conditional on the value of the other variables; thicker edges represent stronger conditional correlations between the nodes, and the symptoms they represent (Borsboom & Cramer, 2013). Edge thickness can be interpreted in terms of the ease with which symptom B (low mood) is activated if symptom A (fatigue) is activated (or vice versa; Borsboom & Cramer, 2013). If the relationship between two nodes in a network is sufficiently strong, then feedback loops between the nodes will lead to sustained activation of the nodes, which is commonly interpreted as modelling a situation in which the sustained interaction of symptoms may lead to the development of mental-health disorders (Borsboom, 2017). Hence, highly connected nodes in a network may represent important clinical targets, as interventions, targeting these nodes, may diminish the spread of activation, thereby reducing the network activation, and presumably in real-world situations the prominence of symptoms that in certain constellations manifest in mental-health disorders (McNally, 2016). Conversely, the absence of an edge indicates that two constructs are conditionally independent, that is, there is no statistical association between these constructs if we fix the value of the other variables (Epskamp & Fried, 2018). If one node is not connected to any other node in the network, then it can be assumed that this node is statistically independent from all other nodes in the network, and likely does not contribute to

the overall network interaction (see Epskamp & Fried, 2018). A central tenet of the network approach is that mental health disorders emerge as a consequence of the sustained interaction between symptoms in strongly connected networks, and dissolve once symptoms deactivate or the relationship between them disappears, which can be visualised in less connected networks (Borsboom, 2017; McNally, 2016).

Statistical analyses

The network estimation was based on the *Ising model* (Ising, 1925; Kindermann & Snell, 1980), which used a series of nodewise logistic regression analyses to estimate the network parameters from our binary data (van Borkulo et al., 2014). The resulting networks were visualised using the R package *qgraph* (Epskamp, Cramer, Waldorp, Schmittmann, & Borsboom, 2012) and the layout '*spring*' (based on the Fruchterman and Reingold (1991) algorithm), whereby the most correlated nodes were placed in the centre of the network, whilst those with the least associations were placed in the periphery (Epskamp et al., 2012).

We estimated the regularised partial correlation network, using the R package *IsingFit* (van Borkulo, Epskamp & Robitzsch, 2016) and the method *elasso* (van Borkulo et al., 2014). Specifically, *elasso* is a regularisation technique that sets small associations to zero, thereby eliminating spurious edges (van Borkulo et al., 2014). We used the default tuning parameter of 0.5 and model selection based on the extended Bayesian Information Criterion (EBIC; Chen & Chen, 2008) to obtain a sparse network (Epskamp & Fried, 2018). This procedure has proven reliable and efficient for the estimation of regularised partial correlation networks from binary data, even in small sample sizes (van Borkulo et al., 2014). By calculating the regularised partial correlation network, we were able to test the unique associations between a) pain and self-harm thoughts and behaviours and b) other correlates of self-harm thoughts and

behaviours, after conditioning on additional nodes in the network and regularisation for weak associations.

Centrality analyses

Furthermore, we performed a series of exploratory analyses to inspect the importance of each node in the network, using the packages *qgraph* (Epskamp et al., 2012) and *networktools* (Jones, 2020) and the R functions *centrality* (Opsahl, Agneessens & Skvoretz, 2010) and *expectedInf* (Robinaugh, Millner, & McNally, 2016). By using this code, we retrieved information on the node's strength and expected influence. In weighted networks, node strength is based on the sum of edge weights and describes the strength with which a given node is connected to other nodes in the network (McNally, 2016). Furthermore, o*ne-step expected influence* describes the extent to which a node influences its direct neighbours, whereas *two-step expected influence* describes the direct and indirect connectivity of a given node up to two edges away from that node (i.e., the cumulative network influence; Robinaugh et al., 2016). As negative edges are taken into account, these expected influence indices were found superior in identifying influential nodes in psychological networks, compared to other commonly studied centrality measures (particularly betweenness and closeness; Bringmann et al., 2019; Robinaugh et al., 2016). Together, these centrality measures assist with the identification of important nodes (Robinaugh, et al., 2016).

Accuracy of network parameters and centrality indices

Using the R package *bootnet 1.2.4* (Epskamp, Borsboom, & Fried, 2018), we tested the accuracy and stability of the estimated network parameters across 1000 bootstrap samples.

Supplement 2. Extra information on the study methodology.

Psychiatric disorders

The Development and Well-Being Assessment (DAWBA; Goodman, Ford, Richards, Gatward, & Meltzer, 2000) is a valid standardised interview package for the assessment of psychiatric disorders in children aged 5 to 16 years (Goodman et al., 2000). The schedule combines structured questions that relate directly to the criteria required to meet psychiatric diagnoses in the International Classification of Diseases, 10th Edition (ICD-10; WHO, 1993) and the Diagnostic and Statistical Manual of Mental Disorders, Fourth Edition (DSM-IV; American Psychiatric Association, 1994), combined with semi-structured probes applied to any areas of difficulty to elicit more detail. There are parallel interviews for parents and for young people aged 11 years or more, with a shorter version for teachers; all three versions were used in the British Child and Adolescent Mental Health Survey (Green, McGinnity, Meltzer, Ford, & Goodman, 2005). The interviews were conducted by lay interviewers while a team of experienced child mental health clinicians combined all available information from all available informants to assign the presence or absence of DSM-IV diagnoses for each child (see Green et al., 2005). When these data were extracted for the main outcome report, reliability statistics on 500 children revealed adequate inter-rater reliability between the two raters (Κ statistic for chance-corrected agreement = 0.86 (any disorder); see e.g., Ford et al., 2017). Hence, we used this integrated information on the presence or absence of psychiatric disorder(s) to obtain the dichotomised variable ‘Psychiatric Disorders’.

Childhood trauma

We used the ‘Post Traumatic Stress Disorder’ module of the DAWBA to determine the presence or absence of childhood trauma (see Green et al., 2005). In this module, parents were

asked to indicate whether their child had experienced any of the following traumatic events; a serious or frightening accident (e.g., a bad car crash), a bad fire (e.g., being trapped in a burning building), other disasters (e.g., earthquake), a severe attack or threat (e.g., by a gang), severe physical abuse that the child still remembers, sexual abuse, rape, witnessed severe domestic violence, saw family member or friend severely attacked or threatened, witnessed a sudden death (e.g., suicide or heart attack), and some other severe trauma, as described by the parents (see Green et al., 2005). The above-mentioned traumatic events were collapsed into the dichotomous variable 'Childhood Trauma', indicating the presence or absence of at least one type of childhood trauma.

Stressful life events

Parents were asked to report whether their child had experienced any stressful life event (i.e., at least <u>one</u> event) from a list of ten events, comprising: involvement in a serious accident, hospital admission, death of a friend or sibling, termination of a close friendship, mental or physical health problems of the parents, parental separation or criminal charges, or major financial crises (see Goodyer, Wright, & Altham, 1990). The above-mentioned stressful life events were collapsed into one dichotomous 'Stressful Life Events' variable, referring to the presence or absence of at least one stressful life event.

Parental mental health

The General Health Questionnaire ([GHQ-12]; Goldberg et al., 1997) is a valid and reliable 12-item self-report questionnaire that was used to measure parental symptoms of depression and anxiety (0: not at all to 1: much more/ less than usual). A cut-off score of at least 3 was used to indicate significant levels of parental distress, which was found to optimally

differentiate between presence and absence of parental distress across 15 centres around the world (Goldberg et al., 1997; Green et al., 2005). Hence, the dichotomised variable 'Parental Distress' refers to the presence or absence of parental distress.

Family functioning

The McMaster Family Assessment Device ([FAD]; Miller, Epstein, Bishop, & Keitner, 1985) is a valid and reliable self-report questionnaire used for the assessment of family functioning. Parents were asked to answer 12-items, using a 4-point Likert-scale (1: strongly agree to 4: strongly disagree). The total score was used to determine general family functioning, with a mean greater than 2 indicating family dysfunction (Miller et al., 1985). Hence, the dichotomised variable 'Family Dysfunction' refers to the presence or absence of family dysfunction.

Peer problems

The Strength and Difficulties Questionnaire ([SDQ]; Goodman, 1997) is a screening questionnaire of childhood psychopathology over the last six months, with proven adequate validity and reliability characteristics across various populations (Goodman, 2001). The 25 items of the SDQ fall within five subscales of each five items: emotional symptoms, conduct problems, hyperactivity-inattention, peer relationship problems and prosocial behaviour (Goodman, 1997).

The peer problem subscale of the SDQ (Goodman, 1997) was used as a measure of the child's peer problems. Using a multiple informant approach, parents, teachers and adolescents (≥ 11 years) themselves were invited to complete the peer problem subscale by scoring each of the five items (0: not true to 2: certainly true). The analyses focussed on the ratings provided

by the parents, as teacher ratings are restricted to school-related difficulties, and self-report ratings were only obtained in children from the age of 11 years onwards. A higher total subscale score accounts for more difficulties with peers, with a score of at least 4 accounting for significant difficulties with peers (Goodman, 1997). Hence, the dichotomised variable ‘Peer Problems’ refers to the presence or absence of peer problems, as reported by the parents.

Inhibitory control deficits

The hyperactivity/ inattention subscale of the SDQ (Goodman, 1997) was used as an ecologically valid measure of the child’s capacity for inhibitory control in daily life. According to the official manual (Goodman, 1997), a total subscale score indicates greater inhibitory control deficits (i.e., teacher/ parent completed SDQ: close to average: score 0-5; slightly raised: score 6-7; high: score 8; very high: score 9-10; self-completed SDQ: Youth in Mind, 2020). Note that across informants a score of at least 6 represents a critical value to differentiate between UK youth with and without inhibitory control difficulties (Goodman, 1997). Hence, a score of at least 6 was used to indicate inhibitory control deficits. We used the dichotomised variable ‘Inhibitory Control Deficits’ to refer to the presence or absence of inhibitory control deficits, as reported by the parents for the reasons described above.

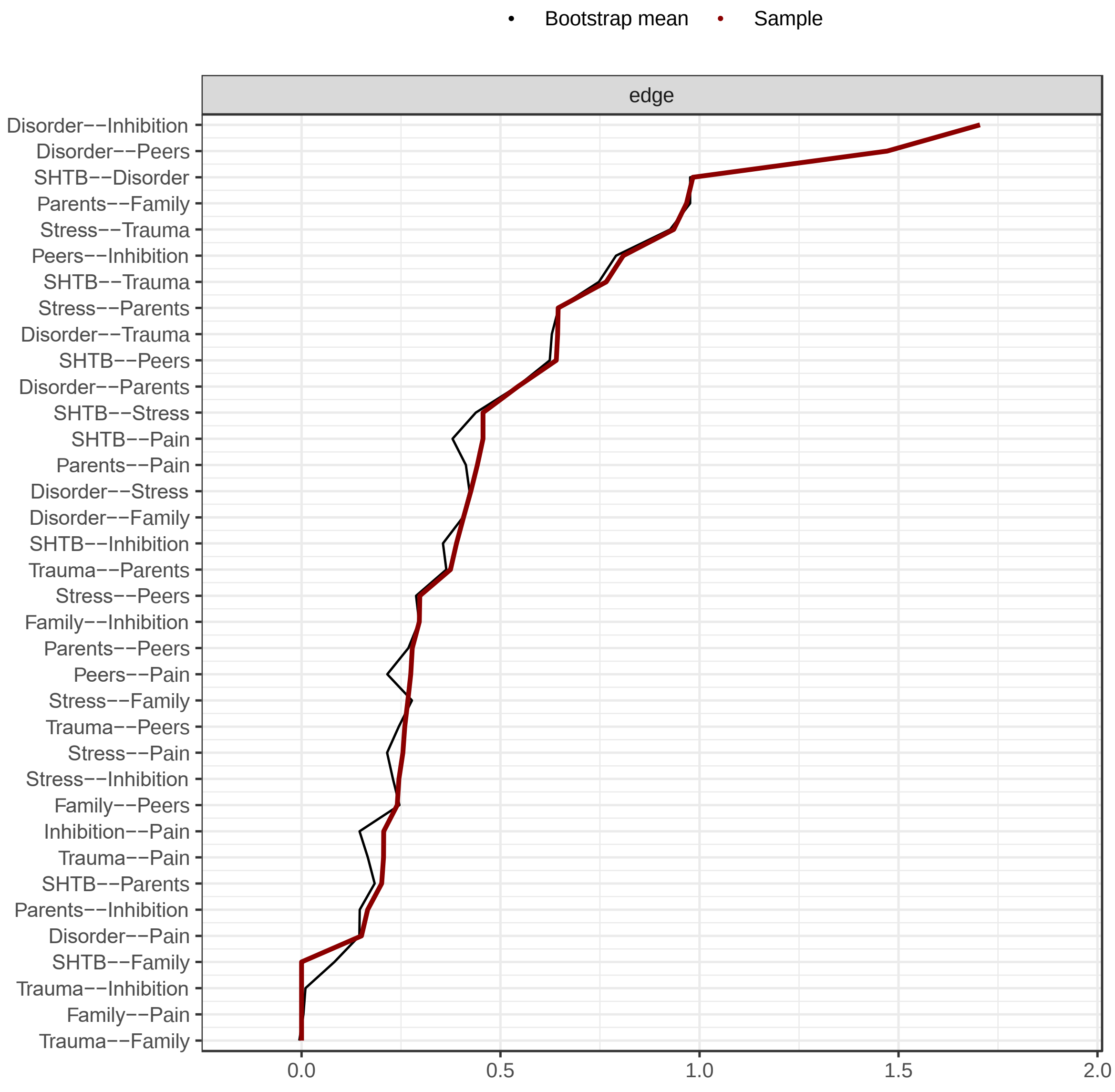

FigS1. Stability of edge weights in the regularised partial correlation network of the whole sample (N=7513).

Note. The confidence intervals were computed over 1000 bootstrap samples. Please note that the bootstrapped confidence intervals for the regularised partial correlation network should only be interpreted based on the width of the confidence interval, with smaller intervals showing stability of edge weight estimates across bootstrap samples, and *not* as a significance test to zero (Epskamp, Borsboom & Fried, 2018).

Legend: SHTB: Self-harm thoughts and behaviours, Disorder=Psychiatric disorder(s), Stress=Stressful life event(s), Trauma=Childhood trauma, Parents=Parental distress, Family=Family dysfunction, Peers=Peer problems, Inhibition=Inhibitory control deficits.

Supplement 3: Network comparison between children and adolescents.

We performed a series of exploratory analyses to compare the regularised partial correlation networks between children (aged 5 to 9 years, *n*=3079) and adolescents (aged 10 to 16 years, *n*=4434; **TableS3A**). The regularised partial correlation networks of both groups showed that the relationship between pain and self-harm thoughts and behaviours was non-significant, after accounting for all other nodes in the network and regularisation for weak associations (**TableS3B**). Whilst, in adolescents, self-harm thoughts and behaviours were most strongly associated with psychiatric disorders, in children self-harm thoughts and behaviours were most strongly associated with childhood trauma. Pain was not associated with any node in the regularised networks of both groups. Furthermore, in both children and adolescents, the variable 'psychiatric disorders' was found to be the most central node in the network, based on all centrality indices (see **TableS3C**). The Pearson correlations between node variability and the centrality indices were non-significant (all $p > .05$), showing that restricted variability across nodes does not influence conclusions regarding the node's importance (see Heeren et al., 2018; Terluin et al., 2016). Consistent with previous studies (see Heeren et al., 2018), expected influence indices were highly correlated in both groups (Children: $r = .99$, $p < .001$; Adolescents: $r = .97$, $p < .001$).

We performed the Network Comparison Test to identify potential statistical differences in the regularised partial correlation networks of children and adolescents. These analyses showed that the regularised partial correlation networks for children and adolescents did not significantly differ in their overall network structure (children=14.02 vs. adolescents=13.51; p=0.484) and network strength (p=0.852). Furthermore, a comparison of edge weights has only highlighted the edges between self-harm thoughts and behaviours and parental distress (p=0.004), as well as between psychiatric disorders and family dysfunction (p=0.018) to be

significantly different in the regularised partial correlation networks of children and adolescents (see also **TableS3B**).

TableS3A. Network estimation for children and adolescents, respectively.

Regularised Partial Correlation Networks

Children

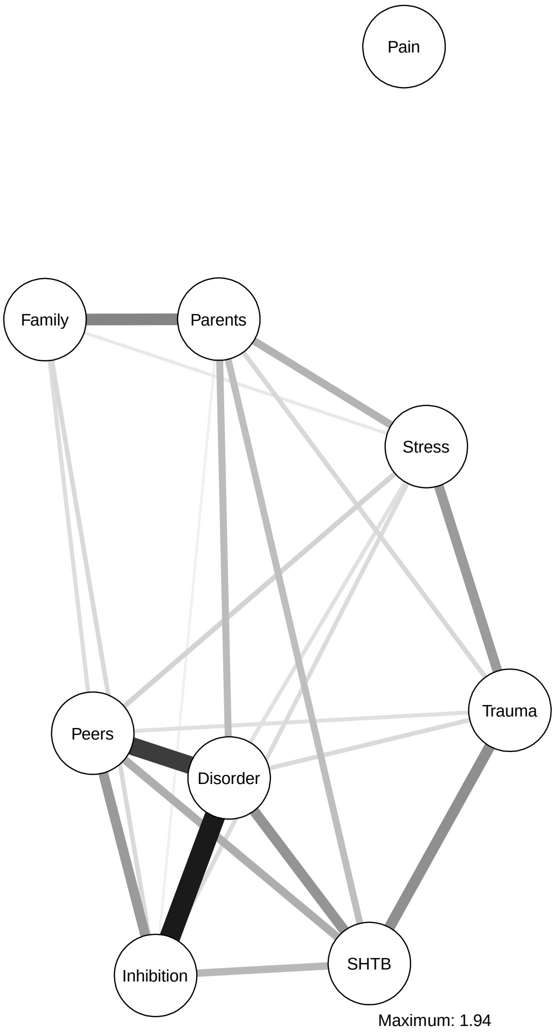

22/36 edges

Adolescents

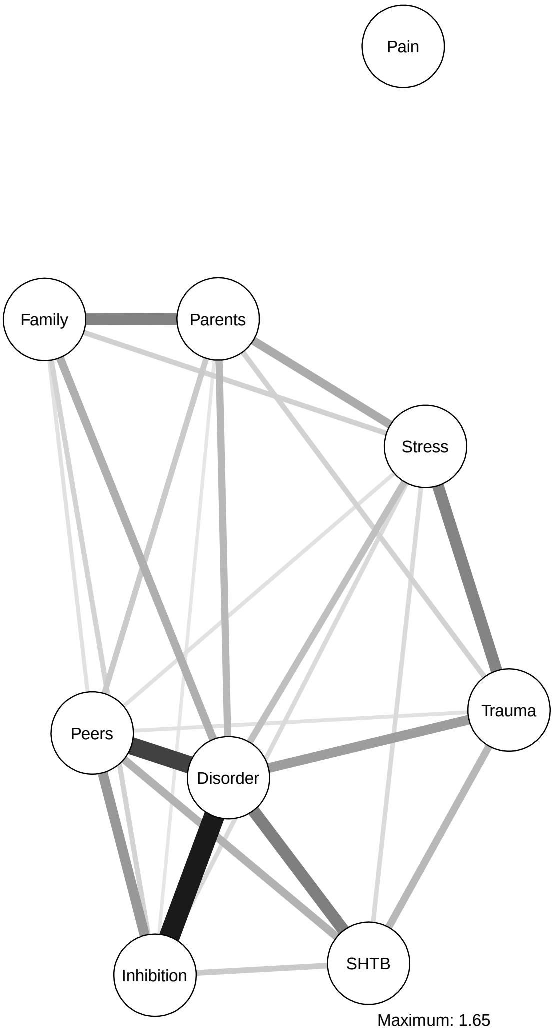

24/36 edges

Note. Children (aged 5-9 years; *n*=3079); Adolescents (aged 10-16 years; *n*=4434).
Legend: SHTB: Self-harm thoughts and behaviours, Disorder=Psychiatric disorder(s), Stress=Stressful life event(s), Trauma=Childhood trauma, Parents=Parental distress, Family=Family dysfunction, Peers=Peer problems, Inhibition=Inhibitory control deficits.

TableS3B. Weights matrices for the regularised partial correlation networks in children (*n*=3079) and adolescents (*n*=4434).

**Children**									
	SHTB	Disorder	Stress	Trauma	Parents	Family	Peers	Inhibition	Pain
SHTB	-	**0.90**	0	**0.95**	0.55	0	**0.69**	0.60	0
Disorder		-	0.26	0.31	0.58	0	1.65	1.94	0
Stress			-	0.85	0.64	0.19	0.37	0.31	0
Trauma				-	0.33	0	0.26	0	0
Parents					-	1.04	0	0.13	0
Family						-	0.29	0.32	0
Peers							-	0.86	0
Inhibition								-	0
Pain									-
**Adolescents**									
	SHTB	Disorder	Stress	Trauma	Parents	Family	Peers	Inhibition	Pain
SHTB	-	**0.92**	0.27	**0.52**	0	0	**0.56**	0.38	0
Disorder		-	0.46	0.71	0.51	0.58	1.37	1.65	0
Stress			-	0.89	0.61	0.33	0.22	0.27	0
Trauma				-	0.33	0	0.21	0	0
Parents					-	0.90	0.38	0.18	0
Family						-	0.22	0.32	0
Peers							-	0.75	0
Inhibition								-	0
Pain									-

Legend: SHTB=Self-harm thoughts and behaviours, Disorder=Psychiatric disorder(s), Stress=Stressful life event(s), Trauma=Childhood trauma, Parents=Parental distress, Family=Family dysfunction, Peers=Peer problems, Inhibition=Inhibitory control deficits.

TableS3C. Centrality analyses for the regularised partial correlation networks in children (*n*=3079) and adolescents (*n*=4434).

**Children**									
	SHTB	Disorder	Stress	Trauma	Parents	Family	Peers	Inhibition	Pain
Strength	3.69	**5.65**	2.62	2.71	3.27	1.84	**4.11**	**4.15**	0
Expected influence									
One-step	3.69	**5.65**	2.62	2.71	3.27	1.84	**4.11**	**4.15**	0
Two-step	18.46	**27.25**	11.64	12.37	13.57	8.27	**21.74**	**22.66**	0
**Adolescents**									
	SHTB	Disorder	Stress	Trauma	Parents	Family	Peers	Inhibition	Pain
Strength	2.65	**6.19**	3.03	2.65	2.90	2.35	**3.70**	**3.54**	0
Expected influence									
One-step	2.65	**6.19**	3.03	2.65	2.90	2.35	**3.70**	**3.54**	0
Two-step	13.96	**25.64**	13.20	12.82	12.90	11.48	**19.15**	**19.60**	0

Note. Higher values show most central (i.e., important nodes).
Legend: SHTB=Self-harm thoughts and behaviours, Disorder=Psychiatric disorder(s), Stress=Stressful life event(s), Trauma=Childhood trauma, Parents=Parental distress, Family=Family dysfunction, Peers=Peer problems, Inhibition=Inhibitory control deficits.

References of the Supplementary Material

American Psychiatric Association (1994). *Diagnostic and Statistical Manual of Mental Disorders (DSM). American Psychiatric Association*: Washington, DC.

Borsboom, D. (2017). A network theory of mental disorders. *World Psychiatry, 16,* 5–13. doi: 10.1002/wps.20375.

Borsboom, D., & Cramer, A. O. J. (2013). Network Analysis: An Integrative Approach to the Structure of Psychopathology. *Annual Review of Clinical Psychology, 9,* 91-121. doi: 10.1146/annurev-clinpsy-050212-185608.

Bringmann, L. F., Elmer, T., Epskamp, S., Krause, R. W., Schoch, D., Wichers, M. . . . Snippe, E. (2019). What Do Centrality Measures Measure in Psychological Networks? *Journal of Abnormal Psychology, 128,* 892-903. doi: 10.1037/abn0000446.

Chen, J., & Chen, Z. (2008). Extended Bayesian information criteria for model selection with large model spaces. *Biometrika, 95*, 759–771. doi: https://doi.org/10.1093/biomet/asn034

Epskamp, S., Borsboom, D., & Fried, E. I. (2018). Estimating psychological networks and their accuracy: A tutorial paper. *Behavior Research Methods, 50*, 195-212. doi: https://doi.org/10.3758/s13428-017-0862-1.

Epskamp, S., Cramer, A. O. J., Waldorp, L. J., Schmittmann, V. D., & Borsboom, D. (2012). qgraph: Network Visualizations of Relationships in Psychometric Data. *Journal of Statistical Software, 48*, 1-18. doi: 10.18637/jss.v048.i04.

Epskamp, S., & Fried, E. I. (2018). A tutorial on regularised partial correlation networks. *Psychological Methods, 23*, 617-634. doi:10.1037/met0000167.

Ford, T., Parker, C., Salim, J., Goodman, R., Logan, S., & Henley, W. (2017). The relationship between exclusion from school and mental health: a secondary analysis of the British Child and Adolescent Mental Health Surveys 2004 and 2007. *Psychological Medicine, 48,* 629-641. doi: 10.1017/S003329171700215X.

Fruchterman, T. M. J., & Reingold, E. M. (1991). Graph drawing by force-directed placement. *Software: Practice and Experience, 21,* 1129-1164. doi: 10.1002/spe.4380211102.

Goldberg, D.P., Gater, R., Sartorius, N., Ustun, T. B., Piccinelli, M., Gureje, O., & Rutter, C. (1997). The validity of two versions of the GHQ in the WHO study of mental illness in general health care. *Psychological Medicine, 27,* 191–197. doi: 10.1017/S0033291796004242.

Goodman, R. (1997). The strengths and difficulties questionnaire: A research note. *Journal of Child Psychology and Psychiatry and Allied Disciplines, 38,* 581-586. doi: 10.1111/j.1469-7610.1997.tb01545.x

Goodman, R. (2001). Psychometric properties of the strengths and difficulties questionnaire. *Journal of the American Academy of Child and Adolescent Psychiatry 40,* 1337–1345. doi: https://doi.org/10.1097/00004583-200111000-00015.

Goodman, R., Ford, T., Richards, H., Gatward, R., & Meltzer, H. (2000). The development and well-being assessment: description and initial validation of an integrated assessment of child and adolescent psychopathology. *Journal of Child Psychology and Psychiatry, 41,* 645–656. doi: https://doi.org/10.1111/j.1469-7610.2000.tb02345.x.

Goodyer, I., Wright, C., & Altham, P. (1990). The friendships and recent life events of anxious and depressed school-age children. *British Journal of Psychiatry, 156,* 689–698. doi: https://doi.org/10.1192/bjp.156.5.689.

Green, H., McGinnity, A., Meltzer, H., Ford, T., & Goodman, R. (2005). *Mental Health of Children and Young People in Great Britain, 2004.* The Stationery Office: London.

Hawton, K., Saunders, K. E., & O'Connor, R.C. (2012). Self-harm and suicide in adolescents. *Lancet, 379*, 2373–2382. doi:10.1016/S0140-6736(12)60322-5.

Ising, E. (1925). Beitrag zur Theorie des Ferromagnetismus. *Zeitschrift fur Physik A Hadrons and Nuclei, 31,* 253–258. doi: https://doi.org/10.1007/BF02980577.

Jones, P. J. (2020, February 13). *Networktools: Tools for Identifying Important Nodes in Networks*. R Package Version 1.2.2.

Retrieved from: https://CRAN.R-project.org/package=networktools.

Kindermann, R., & Snell, J. L. (1980). *Markov Random Fields and Their Applications. Volume 1*. Providence, RI: American Mathematical Society.

Mars, B., Heron, J., Klonsky, E.D., Moran, P., O'Connor, R.C., Tilling, K., … Gunnell, D. (2019). What distinguishes adolescents with suicidal thoughts from those who have attempted suicide? A population-based birth cohort study. *The Journal of Child Psychology and Psychiatry, 60*, 91-99. doi:10.1111/jcpp.12878.

McNally, R. J. (2016). Can network analysis transform psychopathology? *Behaviour Research and Therapy, 86,* 95-104. doi: 10.1016/j.brat.2016.06.006.

Miller, I. W., Epstein, N. B., Bishop, D. S., & Keitner, G. I. (1985). The McMaster's Family Assessment Device: reliability and validity. *Journal of Marital and Family Therapy, 11*, 345–356. doi: https://doi.org/10.1111/j.1752-0606.1985.tb00028.x

Opsahl, T., Agneessens, F., & Skvoretz, J. (2010). Node centrality in weighted networks: Generalizing degree and shortest paths. *Social Networks, 32*, 245-251. doi: https://doi.org/10.1016/j.socnet.2010.03.006

Robinaugh, D. J., Millner, A.J., & McNally, R. J. (2016). Identifying highly influential nodes in the complicated grief network. *Journal of Abnormal Psychology, 125*, 747–757. doi: 10.1037/abn0000181.

Van Borkulo, C. D., Borsboom, D., Epskamp, S., Blanken, T. F., Boschloo, L., Schoevers, R. A., & Waldorp, L. J. (2014). A new method for constructing networks from binary data. *Scientific Reports, 4,* 5918. doi: 10.1038/srep05918.

Van Borkulo, C., Epskamp, S., & Robitzsch, A. (2016, September 7). *IsingFit: Fitting Ising Models Using the ELasso Method.* R package version 0.3.1.
Retrieved from: https://CRAN.R-project.org/package=IsingFit.

World Health Organisation (1993). *The ICD-10 classification of mental and behavioural disorders: Diagnostic criteria for research*. Geneva, Switzerland. Retrieved from: http://www.who.int/classifications/icd/en/GRNBOOK.pdf

Youth in Mind (2020, January 26). *English (UK).* Retrieved from: http://www.sdqinfo.com/py/sdqinfo/b3.py?language=Englishqz(UK)
